# Nuclear magnetic resonance spectroscopy and mass spectrometry data for sulfated isoguanine glycosides

**DOI:** 10.1016/j.dib.2019.105032

**Published:** 2019-12-19

**Authors:** Yuri Uyama, Emi Ohta, Yui Harauchi, Tatsuo Nehira, Hisashi Ômura, Hiroyuki Kawachi, Aya Imamura-Jinda, Mylene M. Uy, Shinji Ohta

**Affiliations:** a Graduate School of Integrated Sciences for Life, Hiroshima University, 1-7-1 Kagamiyama, Higashi-Hiroshima, 739-8521, Japan; b Graduate School of Biosphere Science, Hiroshima University, 1-7-1 Kagamiyama, Higashi-Hiroshima, 739-8521, Japan; c Nagahama Institute of Bio-Science and Technology, 1266 Tamura-cho, Nagahama, Shiga, 526-0829, Japan; d Department of Chemistry, Mindanao State University-Iligan Institute of Technology, Iligan City, 9200, Philippines

**Keywords:** *Bruchidius dorsali*, Pupal case, Sulfated purine alkaloid, NMR, ESIMS

## Abstract

The data presented here are related to the research paper entitled “Rare sulfated purine alkaloid glycosides from *Bruchidius dorsalis* pupal case” [1]. In this data article, we provide 1D and 2D nuclear magnetic resonance (NMR) spectroscopy and electrospray ionization mass spectrometry (ESIMS) data of three undescribed sulfated purine alkaloids, locustoside A disulfate, saikachinoside B disulfate, and saikachinoside A trisulfate isolated from the pupal case of the wild bruchid seed beetle *Bruchidius dorsalis* (Chrysomelidae, Bruchinae) infesting the seed of *Gleditsia japonica* Miquel (Fabaceae).

**Table:** Specifications Table

Subject area	*chemistry*
More specific subject area	*natural products*
Type of data	*Figure*
How data was acquired	*NMR spectroscopy: JEOL A400; ESIMS: Thermo Fisher Scientific LTQ Orbitrap XL mass spectrometer.*
Data format	*Raw and analyzed*
Experimental factors	*The undescribed sulfated purine alkaloids were purified by column chromatography.*
Experimental features	*The isolated compounds were characterized by ESIMS and NMR spectroscopy*
Data source location	*Higashi-Hiroshima, Japan*
Data accessibility	*Data are available with this article*
Related research article	*Y. Uyama, E. Ohta, Y. Harauchi, T. Nehira, H. Ômura, H. Kawachi, A. Imamura-Jinda, M. M. Uy, S. Ohta, Rare sulfated purine alkaloid glycosides from Bruchidius dorsalis pupal case, Phytochemistry Letters 35 (2020) 10–14.*

**Table:** 

**Value of the Data** • The data presents NMR data and ESIMS data of newly isolated sulfated purine alkaloids and could be used by other researchers. • The provided information on the spectroscopic data of sulfated purine alkaloids could be useful for the analysis of spectra and determination of the structure of other sulfated purine alkaloids. • This data can serve as a benchmark for other researchers to elucidate the structures of sulfated purine alkaloids.

## 1 Data

**Fig. 1:**
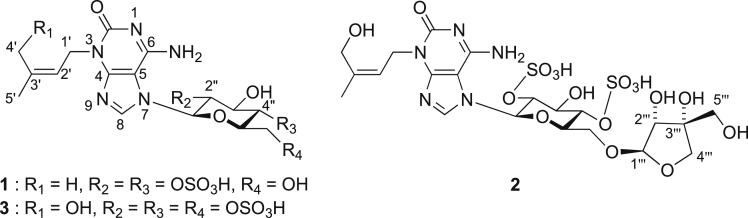
Structures of sulfated isoguanine glycosides isolated from pupal case produced by bruchid beetle *Bruchidius dorsalis* inside *Gleditsia japonica* seeds.

The data set presented in this article focuses on characterization of the sulfated purine alkaloids described in [1]. The article provides the information on the spectroscopic data of the sulfated purine alkaloids **1**–**3** isolated from the pupal case produced by the bruchid beetle *Bruchidius dorsalis* inside the seed of *Gleditsia japonica* (Fig. 1). The ^1^H NMR spectra of **1**–**3** are shown in Fig. 2a, Fig. 3a, Fig. 4a, respectively. The ^13^C NMR and DEPT spectra of **1**–**3** are shown in Fig. 2b, Fig. 3b, Fig. 4b, respectively. 2D ^1^H–^1^H COSY spectra of **1**–**3** are shown in Fig. 2c, Fig. 3c, Fig. 4c, respectively. 2D ^1^H–^1^H NOESY spectra of **1**–**3** are shown in Fig. 2d, Fig. 3d, Fig. 4d, respectively. 2D ^1^H–^13^C heteronuclear single quantum coherence (HSQC) spectra of **1**–**3** are shown in Fig. 2e, Fig. 3e, Fig. 4e, respectively. 2D ^1^H–^13^C heteronuclear multiple-bond correlation (HMBC) spectra of **1**–**3** are shown in Fig. 2f, Fig. 3f, Fig. 4f, respectively. ESIMS data of **1**–**3** are shown in Fig. 2g, Fig. 3g, Fig. 4g, respectively. Analyses of the spectra of **1**–**3** are described in the research article [1]. It has been reported that **3** inhibited starfish blastulation during embryonic development [1].

Although some sulfated guanosine analogs, such as the kainate receptor inhibitor HF-7 [2], have been isolated from the venom of spiders [3], sulfated nucleoside derivatives from natural sources other than spiders are rare [1,4].

## 2 Experimental design, materials, and methods

### 2.1 Samples

Samples were isolated according to a previously reported method [1].

### 2.2 Description of the NMR experiments

Compounds **1**–**3** were dissolved in 0.6 mL of a mixture of CD_3_OD and D_2_O (1:9). All NMR spectra were acquired using a JEOL A400 spectrometer (400 MHz for ^1^H, 100 MHz for ^13^C). NMR analysis was performed using the ALICE2 software (JEOL, Tokyo, Japan). ^1^H and ^13^C NMR chemical shifts were referenced to residual solvent peaks: *δ*_H_ 3.30 (residual CHD_2_OD) and *δ*_C_ 49.0 for CD_3_OD. HRESIMS were carried out using a Thermo Fisher Scientific LTQ Orbitrap XL mass spectrometer at the Natural Science Center for Basic Research and Development (N-BARD), Hiroshima University.

## 3 Sulfated isoguanine glycosides 1–3

### 3.1 6-Amino-7-(2,4-di-*O*-sulfo-β-d-glucopyranosyl)-3,7-dihydro-3-(3-methyl-2-buten-1-yl) -2H-purin-2-one (locustoside A disulfate) (**1**)

1D NMR, 2D NMR, and HRESIMS spectra of the compound **1** are shown in Fig. 2a, Fig. 2b, Fig. 2c, Fig. 2d, Fig. 2e, Fig. 2f, Fig. 2ga–g.

**Fig. 2a:**
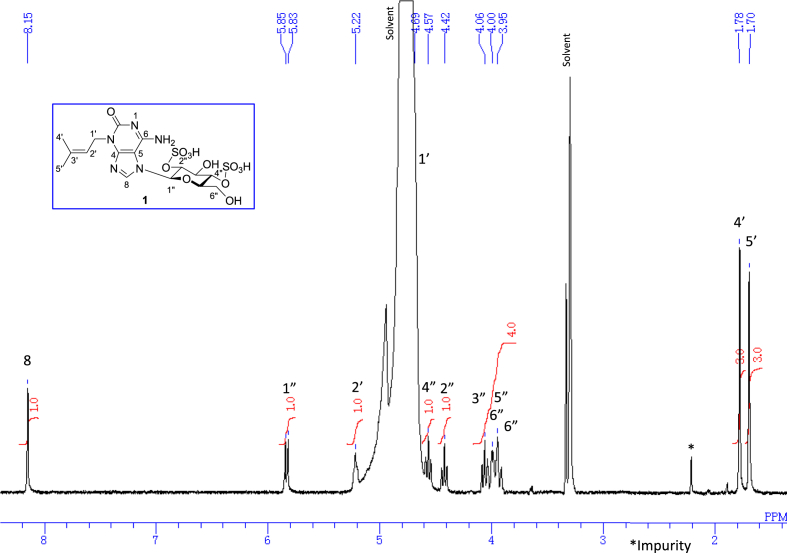
^1^H NMR (400 MHz, CD_3_OD–D_2_O, 1:9) of **1**.

**Fig. 2b:**
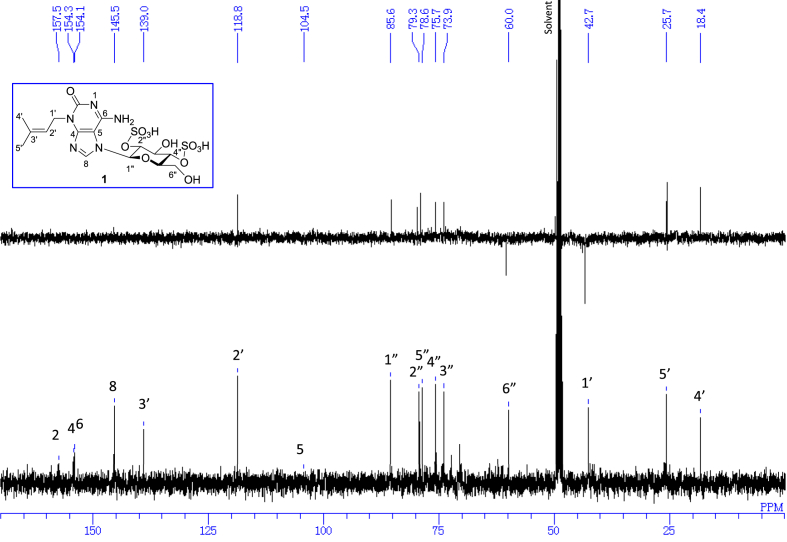
^13^C NMR and DEPT (100 MHz, CD_3_OD–D_2_O, 1:9) of **1**.

**Fig. 2c:**
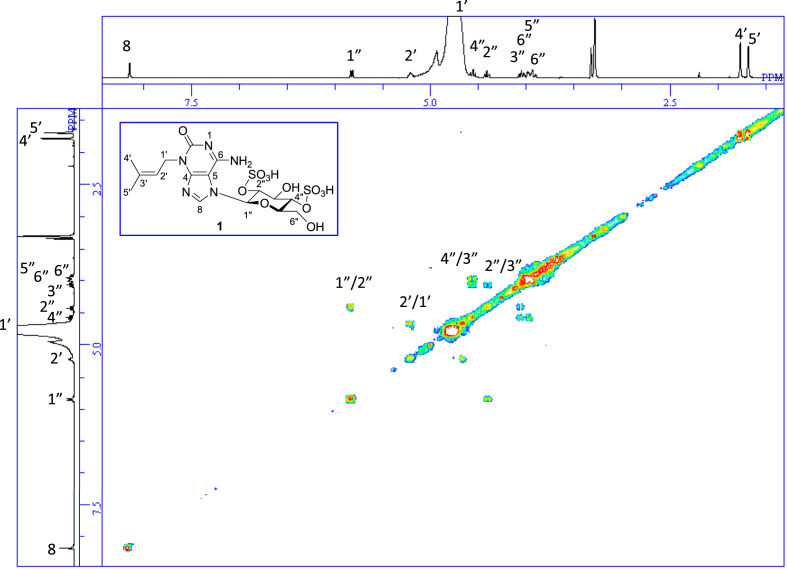
^1^H–^1^H COSY of **1**.

**Fig. 2d:**
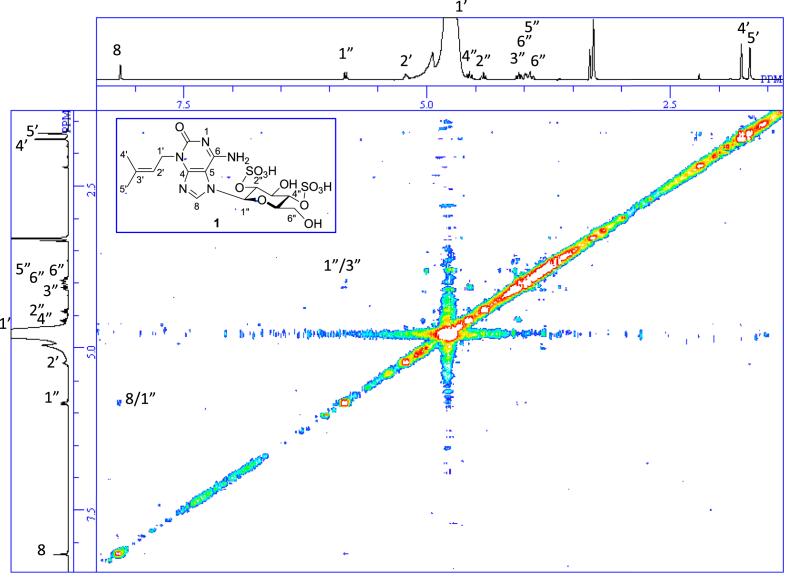
^1^H–^1^H NOESY of **1**.

**Fig. 2e:**
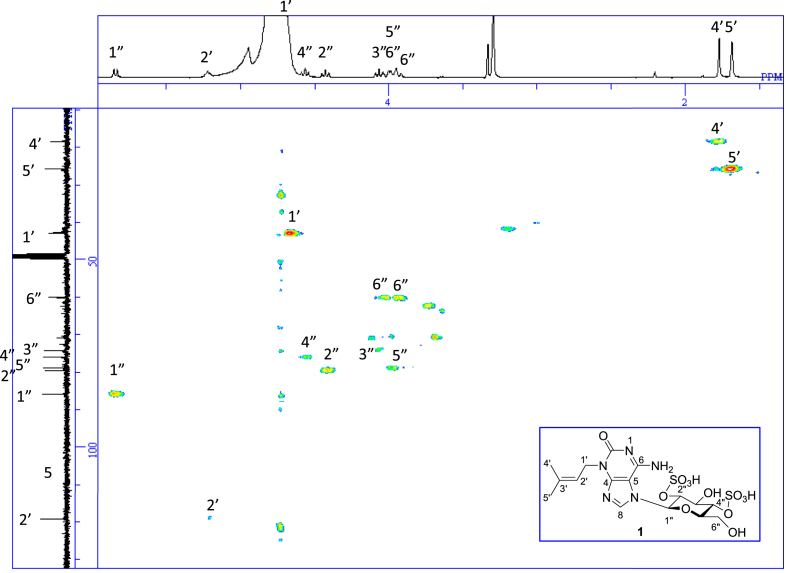
^1^H–^13^C HSQC of **1**.

**Fig. 2f:**
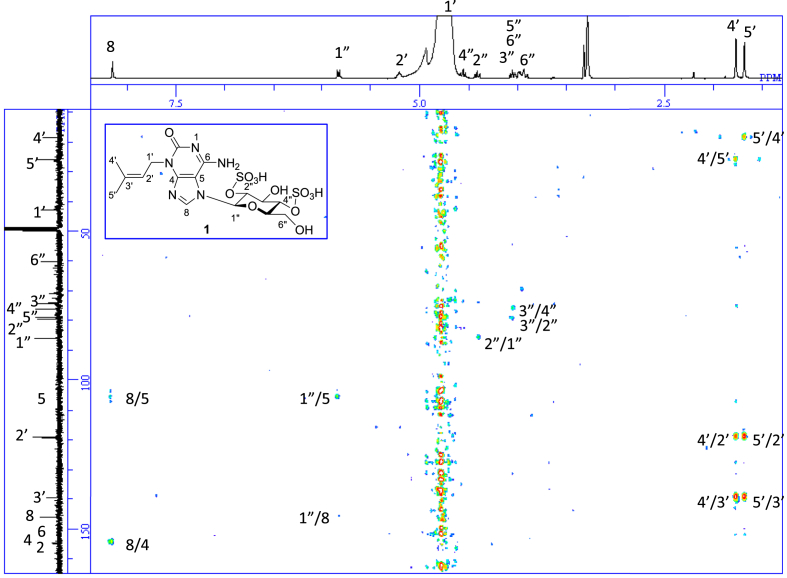
^1^H–^13^C HMBC of **1**.

**Fig. 2g:**
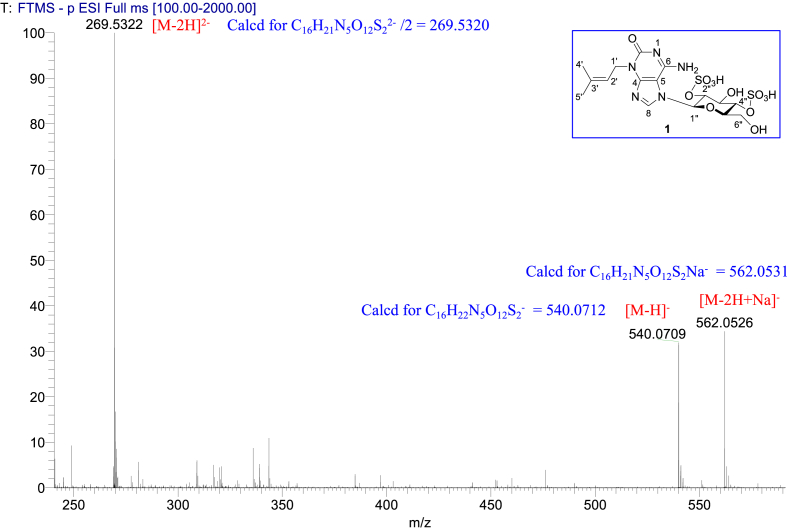
(−)HRESIMS of **1**.

### 3.2 6-Amino-7-(6-*O*-d-apio-β-d-furanosyl-2,4-di-*O*-sulfo-β-d-glucopyranosyl)-3,7-dihydro-3-[(2Z)-4-hydroxy-3-methyl-2-buten-1-yl])-2H-purin-2-one (saikachinoside B disulfate) (**2**)

1D NMR, 2D NMR, and HRESIMS spectra of the compound **2** are shown in Fig. 3a, Fig. 3b, Fig. 3c, Fig. 3d, Fig. 3e, Fig. 3f, Fig. 3ga–g.

**Fig. 3a:**
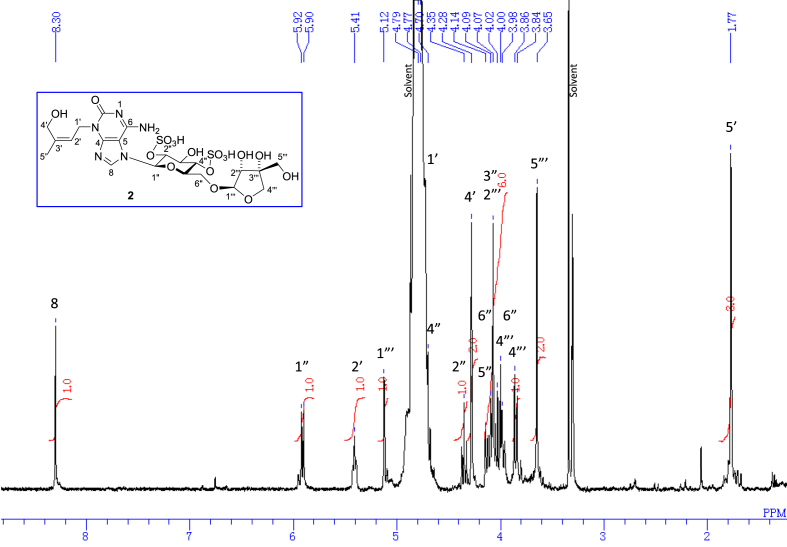
^1^H NMR (400 MHz, CD_3_OD–D_2_O, 1:9) of **2**.

**Fig. 3b:**
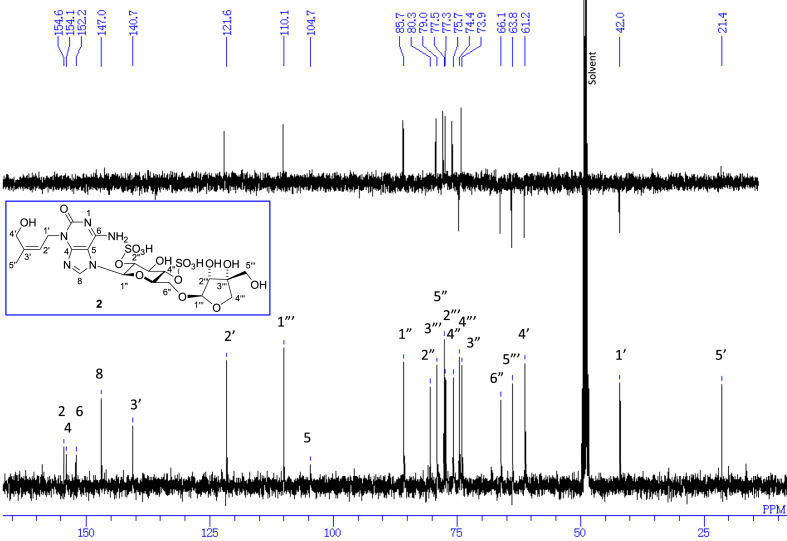
^13^C NMR and DEPT (100 MHz, CD_3_OD–D_2_O, 1:9) of **2**.

**Fig. 3c:**
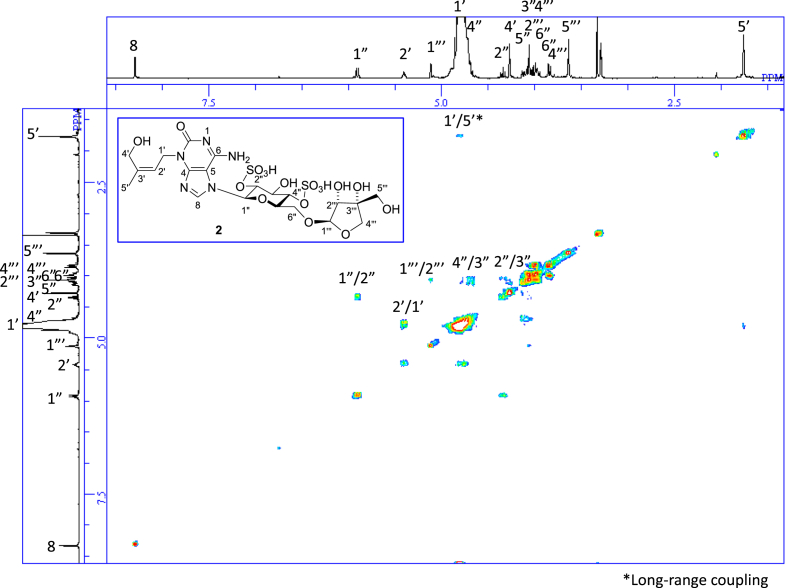
^1^H–^1^H COSY of **2**.

**Fig. 3d:**
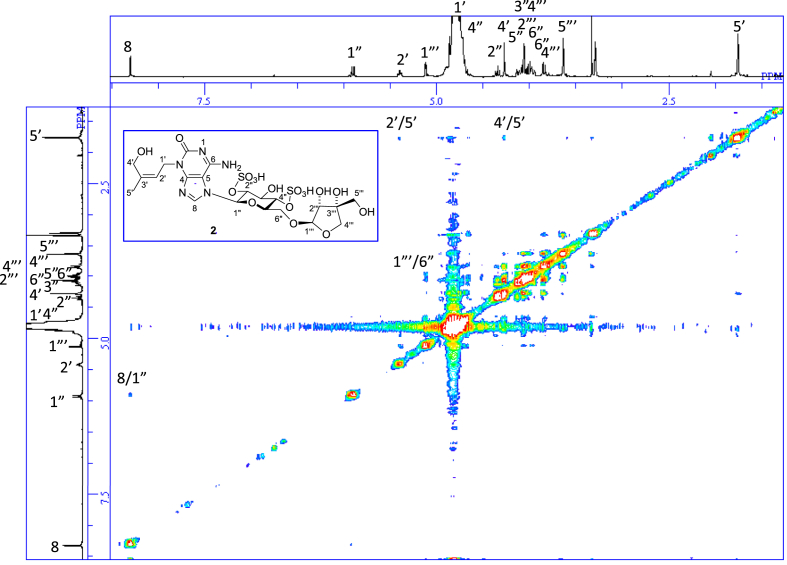
^1^H–^1^H NOESY of **2**.

**Fig. 3e:**
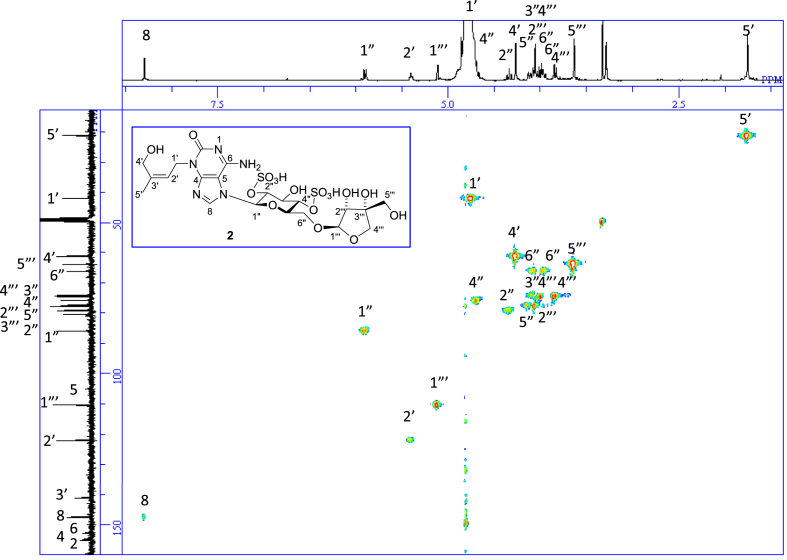
^1^H–^13^C HSQC of **2**.

**Fig. 3f:**
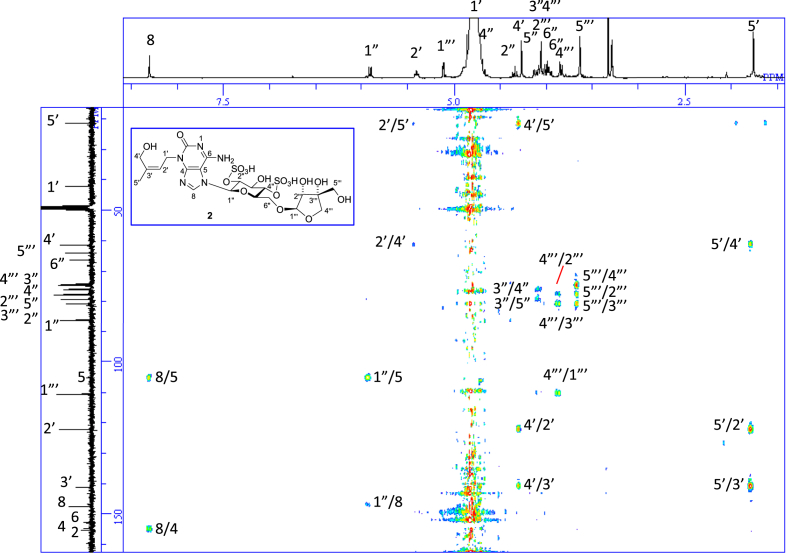
^1^H–^13^C HMBC of **2**.

**Fig. 3g:**
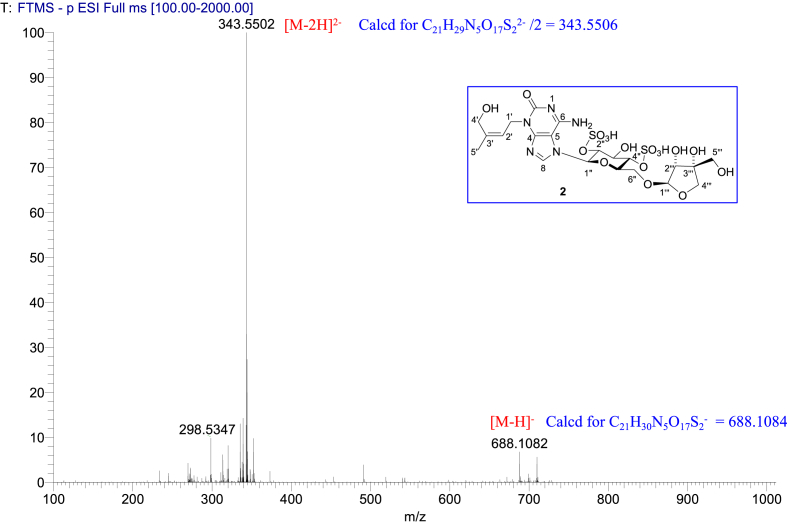
(−)HRESIMS of **2**.

### 3.3 6-Amino-3,7-dihydro-3-[(2Z)-4-hydroxy-3-methyl-2-buten-1-yl]-7-(2,4,6-tri-*O*-sulfo-β-d-glucopyranosyl)-2H-purin-2-one (saikachinoside A trisulfate) (**3**)

1D NMR, 2D NMR, and HRESIMS spectra of the compound **3** are shown in Fig. 4a, Fig. 4b, Fig. 4c, Fig. 4d, Fig. 4e, Fig. 4f, Fig. 4ga–g.

**Fig. 4a:**
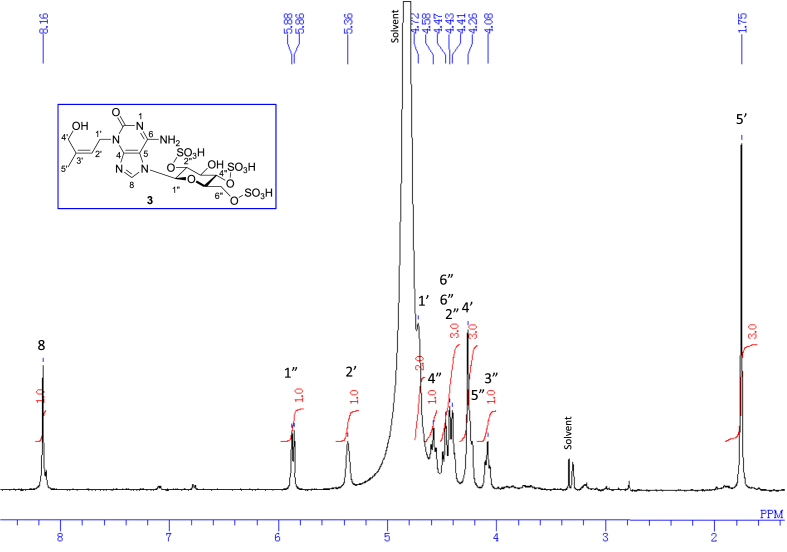
^1^H NMR (400 MHz, CD_3_OD–D_2_O, 1:9) of **3**.

**Fig. 4b:**
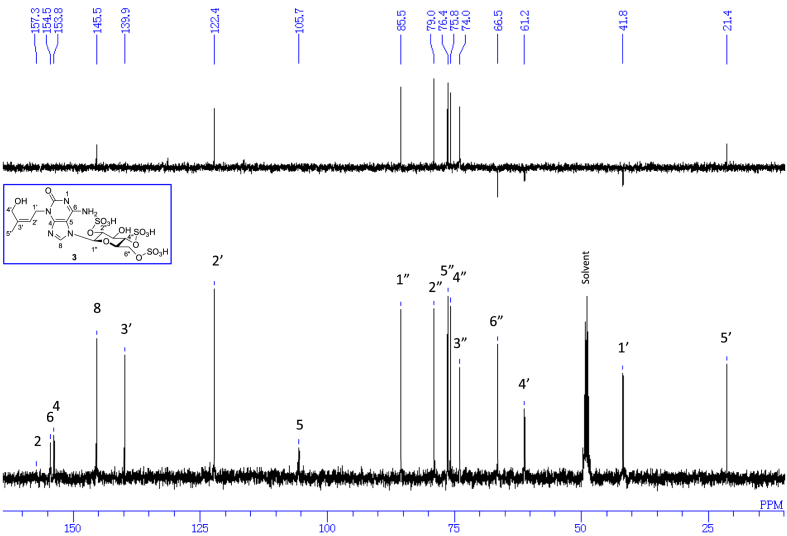
^13^C NMR and DEPT (100 MHz, CD_3_OD–D_2_O, 1:9) of **3**.

**Fig. 4c:**
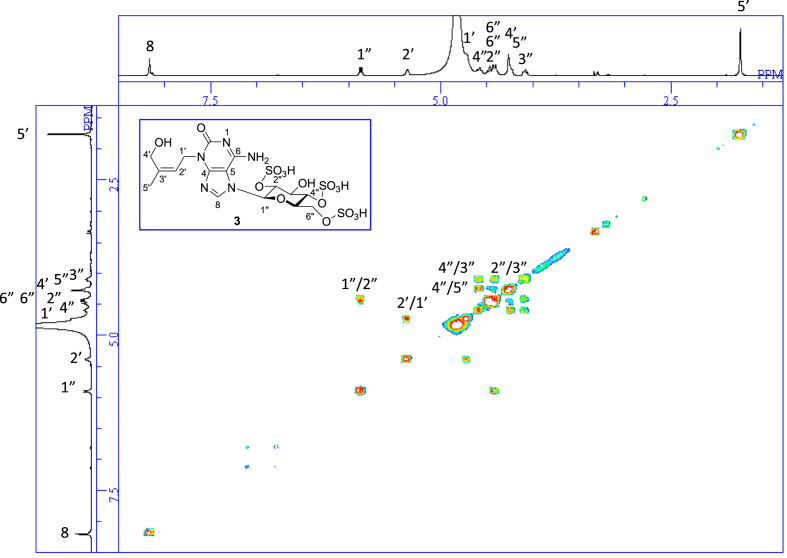
^1^H–^1^H COSY of **3**.

**Fig. 4d:**
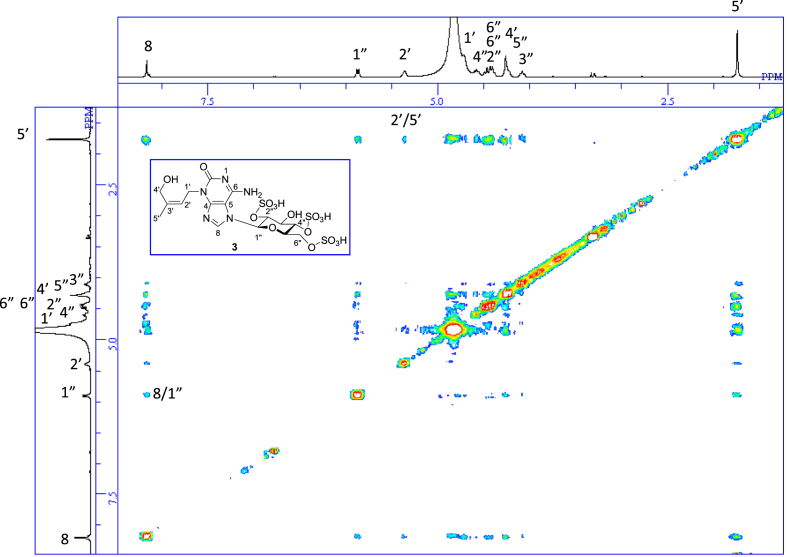
^1^H–^1^H NOESY of **3**.

**Fig. 4e:**
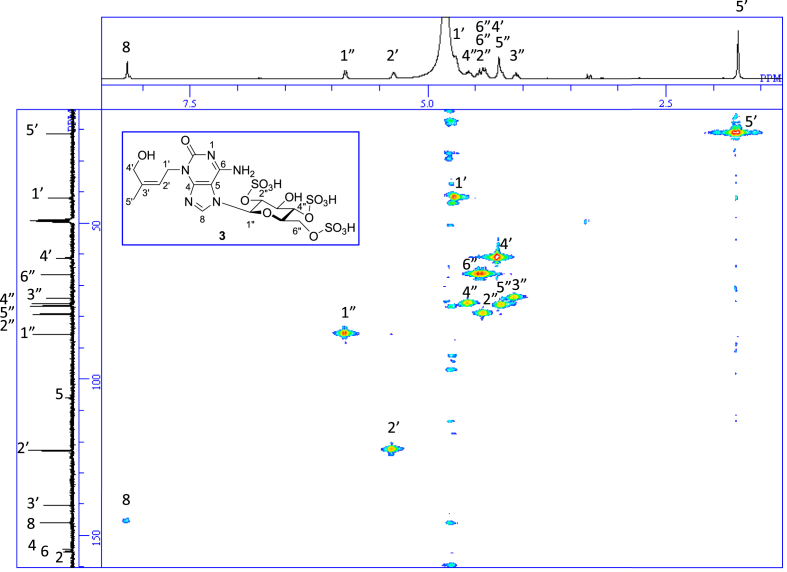
^1^H–^13^C HSQC of **3**.

**Fig. 4f:**
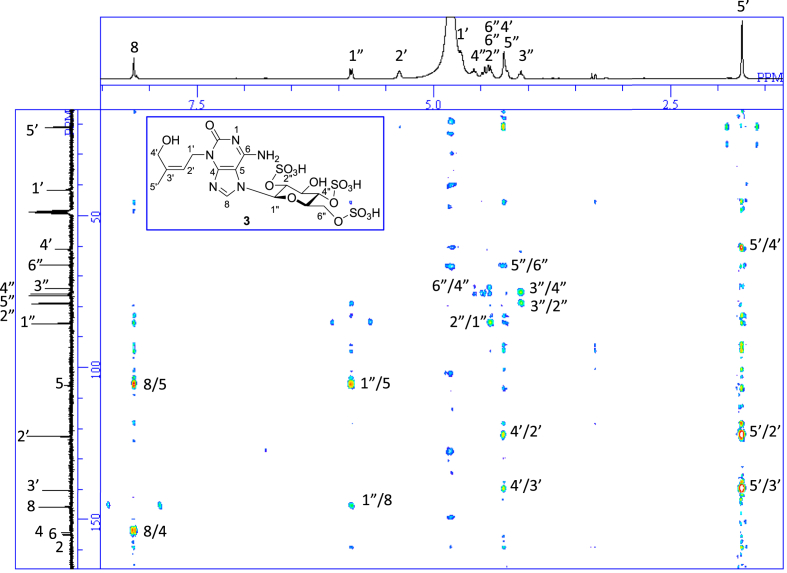
^1^H–^13^C HMBC of **3**.

**Fig. 4g:**
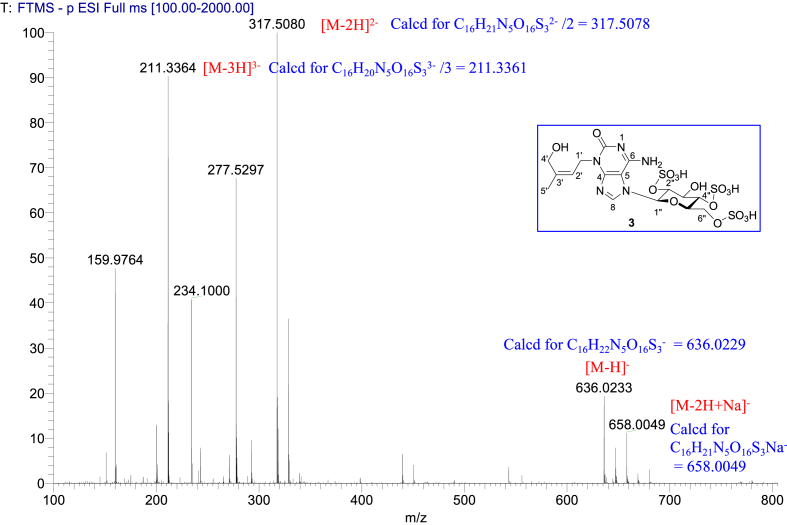
(−)HRESIMS of **3**.
